# Treated together–changed together: The application of dyadic analyses to understand the reciprocal nature of alliances and couple satisfaction over time

**DOI:** 10.1111/jmft.12595

**Published:** 2022-05-23

**Authors:** Kristoffer J. Whittaker, Sverre Urnes Johnson, Ole André Solbakken, Terje Tilden

**Affiliations:** ^1^ Research Institute, Modum Bad Psychiatric Center Vikersund Norway; ^2^ Psychological Institute University of Oslo Oslo Norway

**Keywords:** clinical evidence‐based, outcomes, populations assessment/diagnosis, research couples, research process, theory/model

## Abstract

In a Norwegian study of 73 couples attending a residential couple therapy program lasting between 6 and 12 weeks, weekly self‐report data on therapy alliance and couple satisfaction were collected using routine outcome monitoring (ROM). The aim was to show how dyadic analyses could be applied to examine the predictive association between alliances and couple satisfaction. Results showed that improved alliance between dyad members and their couple therapist predicted their spouses' couple satisfaction. Furthermore, improved couple satisfaction predicted improvement in spouse's alliance. The clinical implication of these findings should heighten awareness to the importance of establishing and maintaining the alliance of male partners in couple therapy, something that predicts their spouses' couple satisfaction. These findings help nuance the already existing literature on the working alliance. Furthermore, we propose that dyadic analyses should be widely used in any psychotherapeutic research that aims to understand the reciprocal effects of dyads.

A major limitation of quantitative research conducted within the field of couple therapy is that it has been done almost exclusively at the individual level of analysis. The individual level refers to changes in self‐perceived intrapsychic or interpsychic functioning which is analyzed without taking the reciprocal nature of relationships into account (Kenny et al., 2020). Of particular interest to researchers and practitioners of couple therapy are effects of reciprocity at the dyadic level (e.g., increased relationship satisfaction) or family level (e.g., improved family functioning; Gurman & Kniskern, 1978). The approach of solely considering the individual level ignores reciprocal effects (i.e., how dyad members' behaviors influence on one another) thus constituting a restriction because it only reveals how an individual respondent changes from one point of assessment to another. Although this is a known limitation within the field of couple therapy research it is either dealt with by handling quantitative data insufficiently or by shunning such methods altogether (Ochs et al., 2020). Examples of the former are averaging outcome scores between dyad members or running separate analyses for husbands and wives. In both these examples one does not account for interdependence between scores (i.e., nested data) and as such the phenomenon of interest, that is, the reciprocal nature of relationships, is omitted as the object of research (Kenny et al., 2020).

In this article, we aim to present an alternative to analyzing data at the individual level and we will be doing so by investigating the associations between therapeutic alliance (i.e., the emotional bond between the therapist and client, and their agreement upon the tasks and goal of therapy; Bordin, 1979) and couple relationship satisfaction. Even though a large body of literature has been dedicated to this topic, demonstrating that the strength of the alliance between the therapist and the client is predictive of the treatment outcome (Del Re et al., 2021; Flückiger et al., 2018; Friedlander et al., 2018), our study objective is to further nuance such findings by showing how dyadic analysis (Kenny et al., 2020) can be applied to identify reciprocal effects across time.

Dyadic analyses (Kenny et al., 2020) is a methodological adaptation to already existent statistical approaches such as mixed modeling (MM; Curran & Bauer, 2011) or structural equation modeling (SEM; Weston & Gore, 2006). As the name of the methods collectively known as dyadic analysis implies, it shifts the level of analysis from the individual to the dyadic. Dyadic analyses are applicable to data collected at one or several timepoints to inquire into a suggested covariation between dyad members. The use of dyadic analyses as a tool to analyze longitudinal data is of great interest. Such longitudinal studies have the potential of identifying moderators and mediators (i.e., processes) of treatment outcomes across time (Kazdin, 2007). As the implementation of routine outcome monitoring (ROM; Tilden & Wampold, 2017) is becoming standard practice within many clinics, the generation of frequently collected data has soared. Such data are applicable within longitudinal research designs to increase our understanding of how systems change over time. In the present study, we are examining how reciprocal effects evolve over time and have thereby chosen to conceptualize the nested nature of dyadic data within the framework of the actor‐partner interdependence model (APIM; Cook & Kenny, 2005). In the APIM reciprocal effects are named actor‐partner effects (Cook & Kenny, 2005). See Figure 1 for a visual representation of the APIM. If instead change is assumed to occur in a deterministic fashion a dyadic growth curve model may be suitable. We refer to Kenny et al. (2020) for an in‐depth discussion on how to select the best fitting model for longitudinal dyadic datasets.

**Figure 1:**
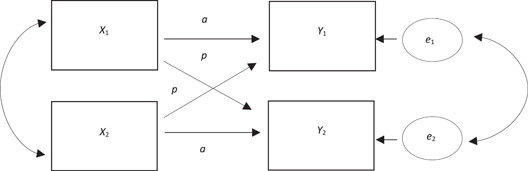
APIM for overtime data. *X* = process variable; *Y* = outcome variable; *a* = actor effect; *p* = partner effect; *e* = error. In a cross‐lagged design, this pattern is repeated with *Y* variable being lagged one timepoint after the *X* variable. The presented model is adapted from Kenny et al. (2020).

Contrary to the central assumption in linear quantitative methods—that observations (i.e., data) are independent from one another (Kenny et al., 2020), the fundamental assumption of the APIM is nonindependence of data. Nonindependence proposes that people who are in the same condition, such as a couple attending therapy together, would have interdependent outcome scores. Thus, their behaviors during therapy (e.g., their alliances with their couple therapist) may not just predict their own outcome (e.g., relationship satisfaction) but also the outcome of their partner. Dyadic analysis allows for testing of such assumed reciprocal effects, that is, a test of nonindependence.

Nonindependence may originate from different sources such as compositional effects (e.g., similarity in personality; Klohnen & Luo, 2003), common fate (e.g., shared contextual factors; Ledermann et al., 2010) and actor‐partner effects (e.g., effect of husbands depression on spouses marital satisfaction; Kenny, 1996; Kenny et al., 2020). Compositional effects are not of particular interest to couple therapy researchers as they likely represent traits that are less malleable and thus do not greatly impact how such systems change over the timespan of therapy (Kenny, 1996). In the following, we will only be discussing actor‐partner effects as a source of nonindependence within the APIM. For a detailed discussion on when to choose the common fate model rather than the APIM, we refer to Galovan et al. (2017) for further reading.

As can be inferred from several meta‐analyses within the couple therapy research literature (Rathgeber et al., 2019; Roddy et al., 2020; Shadish & Baldwin, 2003; Shadish & Baldwin, 2005)—a vast majority of studies applied research designs neglecting the investigation of reciprocal effects. At best such studies show how individuals change from one point to another (usually just two timepoints) in accordance with the nomothetic approach (i.e., with the objective of making general predictions about the population; Beltz et al., 2016). They do not divulge how dyads change across time and are thereby of limited interest beyond illustrating the general efficacy of treatments. Although there exists a growing body of research that applies dyadic analyses to study reciprocal effects, most of them are within the fields of social psychology, and family and developmental studies (Kenny et al., 2020). Despite the benefits of dyadic analyses being apparent, its application in couple therapy research has to the authors' knowledge so far been sparse.

Anderson and Johnson (2010) seminal paper on therapeutic alliances was most likely the first scientific publication to introduce the APIM to the field of couple therapy. A common drawback of the studies conducted (e.g., Anderson & Johnson, 2010; Anderson et al., 2020; Bergeron et al., 2020)—is that they include a limited set of assessment points. Essentially, this means they reveal reciprocal influences, but not across time. In our present study, we aim to show how dyadic analyses may be used in a longitudinal design to address therapeutic alliances and relationship satisfaction as the objectives measured frequently throughout the course of treatment. Hence, we examine how members of dyads reciprocally influenced each other when they conjointly attended intensive inpatient couple therapy. The high frequency of assessment points and juxtapositioning of the outcome variable with the process variable was done with the intention of further investigating the link between alliances and relationship satisfaction. Hence the goal of this study is twofold, both echoing the purpose of Anderson and Johnson (2010) pioneering work and expanding upon their research questions. First, we will do so by familiarizing readers with dyadic analyses and how it may be applied by using MM and conceptualized within the APIM to study processes and outcomes in couple therapy. MM was chosen as it is considered the most flexible approach to estimating the APIM (Kenny et al., 2020). Second, we will advance knowledge of how therapist‐client alliances are associated with relationship satisfaction across time in the context of inpatient couple therapy. In accordance with this latter aim, we will investigate the following research questions:

1. Does the individual's alliance with the therapist predict his or her own couple relationship satisfaction across time? (Actor effect).
2. Does the individual's alliance with the therapist predict their partner's couple relationship satisfaction across time? (Partner effect).
3. Does the individual's couple relationship satisfaction predict his or her own alliance with the therapist across time? (Actor effect).
4. Does the individual's couple relationship satisfaction predict the alliance of their partner with the therapist across time? (Partner effect).
5. Are any actor and partner effects influenced by gender? (Gendered).

## METHODS

### Ethical approval

The authors of this article applied for ethical approval of this study. The subsequent approval was granted by the Regional Ethical Committee (REFNUM 2018/148).

### Treatment

This is a naturalistic study of couple therapy in a residential clinic within the Norwegian Public Healthcare system, the Family Unit (FU) at Modum Bad Psychiatric Center, located in Vikersund, Norway. Due to public health insurance treatment is free of charge. Couples stayed at the FU for approximately 6–12 weeks. They were referred to the FU by their general practitioner, often in collaboration with local mental healthcare providers. Although children ranging from 1–16 years of age also accompany their parents during hospitalization, they are not actively involved in therapy and are therefore not further discussed. The criteria for hospitalization were that at least one member of the couple was diagnosed with a mental disorder according to the International Classification of Disease 10th ed. (ICD‐10; World Health Organization, 1992) and that they suffered from coexistent relational distress (e.g., extensive verbal abuse, problems with intimacy, the stress of parenting, or extramarital affairs). All who applied for hospitalization at the FU were interviewed by staff about their psychiatric and relational histories to assess if the treatment program was suitable for them. Before considering hospitalization at the FU, it was assessed that prior treatment provided by local mental healthcare was unsuccessful. All hospitalizations at the FU were planned. People who were actively suicidal, psychotic, or had ongoing substance abuse were not admitted to the FU, and neither were couples with ongoing interpersonal physical violence.

The FU has a total of 12 therapists (of whom 66.67% were women, with the average age of 44.58, and with 7.16 years average length of basic and ongoing education as mental healthcare professionals) servicing each of the nine couples committed at any given time. All the couples were treated by at least two therapists (either a clinical psychologist, psychiatrist, or family therapist coupled with a specialist nurse and/or family therapist). A medical doctor examined all patients at intake. Couple therapy as applied within this unit should be understood as an integrated part of a comprehensive treatment program, thus comprising a greater variety of treatment components than what is common within regular outpatient couple therapy services. Such components include semiweekly couple therapy sessions (e.g., emotionally focused, psychodynamic, and collaborative), a weekly art therapy session, a weekly psychoeducation session, and semiweekly physical exercise sessions. The treatment program offered at the FU although targeting the couple relationship does not adhere to a specific therapeutic model but is best understood as the integration of the systemic and individual perspectives. Thus, a range of intervention strategies applied was drawn from individual, couple, and family therapy models. For a detailed description of the treatment program, we refer to Tilden (2008). All therapists participated in a biweekly peer‐counseling with an external supervisor, and weekly supervision making use of the information from patients' structured feedback with an internal supervisor. As this project was naturalistic and plural, non‐manualized therapeutic approaches were applied, thus no adherence to any specific couple therapy model was monitored.

### Participants

All patients hospitalized at the FU between January 2018 and April 2021 were eligible for inclusion. Of the 196 patients invited to participate in the current study, 169 gave their informed consent. A further 23 were excluded because they did not meet the inclusion criteria, either because they were not hospitalized with their spouse or because they were not in a heterosexual relationship. Thus, 146 individuals constituting 73 heterosexual couples made up the present sample. The mean age was 40.99 years (SD 7.56, range 24–61). A total of 74.7% of the participants were either before or during hospitalization diagnosed with a psychiatric disorder, 23.7% fulfilled the criteria for two diagnoses, while 4.8% fulfilled the criteria for three diagnoses. The most common category of diagnoses was affective disorders (34.2%) and adjustment disorders (33.5%) including posttraumatic stress disorder (PTSD). A significant higher proportion of women (83.5%) had such diagnosis than men (65.7%, *χ* ^2^, *p* = 0.014).

Of the 146 participants, 82.1% (*n* = 120) had completed a form on medication use during treatment. Of these respondents, 34.9% reported the use of medication at intake (18.3% anti‐depressants, 8.3% analgesics, 5% antianxiety, and 3.3% hypnotics). A further 12.5% reported using a second medication (7.5% hypnotics, 3.3% antianxiety, and 1.7% analgesics), while 5.8% of these participants used three or more medications (3.3% hypnotics and 2.5% analgesics) at the time of hospitalization. At the end of treatment, 32.5% reported using a medication. The use of a second medication had been reduced to 9.2%, while the use of three or more medications had been reduced to 5%. However, these reductions from pretreatment to posttreatment on medication use both in general as well as the use of a second medication (from 34.9% to 32.5%, and from 12.5% to 9.2%, respectively) were nonsignificant (*χ* ^2^ 0.15, *p* = 0.69 and *χ* ^2^ 0.67, *p* = 0.41, respectively).

Of those participants that had completed, the form on medication use (*n* = 120), 18.3% reported to their therapist that they had been the victim of sexual abuse during childhood, while 19.7% had been exposed to childhood physical abuse, and a further 48.3% reported having experienced other traumatic events during childhood. More than half (54.6%) of the respondents had experienced incidents of repeated trauma during childhood. Of the households (*n* = 60) represented by these respondents, 41.6% had an adult family member (i.e., parent or spouse) who had been exposed to repeated traumatic events during childhood. For exposure to repeated traumatic events during the adulthood, the amount households affected are 36.6%. A number of participants out of the these 82.1% had also been forthcoming in divulging histories of addiction (15.8%), and/or self‐harm (14.2%), and/or attempts at suicide (7.5%).

### Procedure

Systematic collection of frequent measurements and their application as feedback in therapy sessions, supervision, and data in research has been a longstanding practice at the FU. Frequent measurements were conducted every week during hospitalization. All questionnaires were self‐report and completed online.

### Weekly measures

The Revised Dyadic Adjustment Scale (RDAS; Busby et al., 1995) is a widely used 14‐item questionnaire providing a global measure of each partner's assessed consensus, satisfaction, and cohesion toward their spouse. The scoring range is 0–69 with higher scores representing better adjustment and with 48 as a cutoff. The RDAS shows acceptable psychometric properties (Busby et al., 1995). Cronbach's alpha was at admission 0.81 and 0.85 at end of treatment.

The Working Alliance Inventory (WAI; Horvath & Greenberg, 1989) is a widely used questionnaire to assess the therapeutic alliance between the therapist and client. Seven items (two goal, two task, and three bond‐related questions) from the WAI were included in the battery of questionnaires that the participants completed weekly. Studies on short versions of the WAI have demonstrated good psychometric properties (Hatcher & Gillaspy, 2006; Munder et al., 2010). Higher scores on the WAI represent a stronger working alliance. Cronbach's alpha was at admission 0.88 and 0.90 at end of treatment.

### Statistical analyses

The data set includes frequent assessments from the beginning of treatment until the end of treatment and was analyzed as a two‐level structure (weekly observations of participants nested within their respective dyads) using longitudinal MM (Curran & Bauer, 2011). With up to 12 measurement waves the data set meets the requirements proposed by Singer et al. (2003) for the application of MM. To be able to perform a dyadic data analysis the data had to be organized in a pairwise data structure as suggested by Kenny et al. (2020). The pairwise restructuring also resulted in a long‐format data set—a prerequisite for longitudinal data analysis. A webinar created by West and Thorson (2017a) gives a practical guide on how to restructure from an individual format to a pairwise format.

Unlike traditional models for frequent measures, MM's can effectively manage missing data, because it is based on maximum likelihood (ML). ML is considered “state of the art” for handling missing data (Schafer & Graham, 2002). ML uses all the available data, complete and incomplete, to identify the parameter values that have the highest probability of producing the sample data. Thus, all 73 dyads participating in the study were included even if data was partially missing. Research indicates that ML is preferable to multiple imputations when using longitudinal data (Shin et al., 2017).

A dummy variable (gender) was construed for distinguishing between two members from the same dyad. The primary reason for using gender as the distinguishing variable instead of other variables of interest such as the presence of a psychiatric disorder, was because it granted the highest possible sample size and allowed for interpreting the data within a gender framework. More importantly, the inclusion of a dummy variable permitted the assessment of the explained variance of each dyad member's estimated scores and to what extent these scores correlated. The resulting correlation coefficient is our measure of nonindependence (Kenny et al., 2020).

All process variables were mean centered. In our first analyses, the WAI constituted the process variable and the RDAS was used as the outcome variable. We then reran the analyses by applying the RDAS as the predictor variable and the WAI as the outcome variable. This was done to investigate other patterns of influence than those generally assumed in the literature on the working alliance (i.e., the alliance as a predictor of outcome; Flückiger et al., 2018; Friedlander et al., 2018).

Timepoints were centered, and a dummy variable we called obs_id was construed to give every dyad a unique id for every timepoint. We time‐lagged the mean‐centered process variables to allow for cross‐lagged regressions analysis for the estimation of how scores from one timepoint predict the estimated scores at the following timepoint. The time‐lag procedure we adhered to is described by West and Thorson (2017b). The APIM served as the conceptual model for the cross‐lagged regressions analysis. The equation for this model is:

$$Y_{1ti}=c_{1i}+a_{1i}Y_{1,t-1,i}+p_{12i}Y_{2,t-1,I}+e_{1ti}$$

$$Y_{2ti}=c_{2i}+a_{2i}Y_{2,t-1,i}+p_{12i}Y_{1,t-1,I}+e_{2ti}$$

In our analysis, *i* represents the dyad while the *Y's* represents the assessed variable of each dyad member at timepoints *t* and *t* −1 (i.e., time‐lagged by 1). The *a's* represent the actor effects and the *p's* represent partner effects, while *e* represents the error terms for each member of the dyad. The intercept of each dyad member is denoted by *c*_1_ and *c*_2_ respectively. According to our equation, there are six parameters (two actor, two partner, and two intercepts) that may vary across dyads as well as covary with each other. The two error terms (one for each dyad member) are of special importance as they potentially correlate. This correlation between error or *r*_ee_, measures to which extent the two members of a dyad are similar or not from one timepoint to another (i.e., assessment of nonindependence).

Following the steps proposed by Kenny et al. (2020), we were able to analyze our data within a cross‐lagged framework in SPSS v. 27. Kenny et al. (2020) approach to dyadic cross‐lagged regressions analysis recommends starting modelbuilding with a fully saturated model, simplifying the model if it does not run. In our study's model, the random effects were removed. This decision was made because the results after applying random effects were not a definitive positive even though all convergence criteria were satisfied. This implied that the validity of the results could not be ascertained. On further inspection of the output, it became clear that the random effects were either not significant or were otherwise considered redundant. Running the model without random effects resulted in no further hindrances. Thus, their removal resulted in a more parsimonious model. The applied covariance structure CSH (compound symmetry: heterogenous) produces separate error variances for each dyad member, which is central to establish the degree of nonindependence between member scores. We also implemented test statements which are necessary to assess if differences between dyad members were significant. Thus, the results from the test statements further controlled for gender allowing for easier interpretation of the results (i.e., whether the one effect is stronger than the other). We refer to a webinar by David Kenny (2015) for a step‐by‐step walkthrough on how to build a MM including test statements and interpreting the output.

## RESULTS

### Descriptive statistics

See Table 1 for descriptive statistics of the included variables on the individual level of assessment. See Table 2 for Pearson's correlations of the included variables on pre‐ and posttest assessments.

**Table 1:** Descriptive statistics on the individual level.

Instrument	Type of test	*N*	*M* (*SD*)	Range
RDAS	Pretest	136	39.48 (7.85)	20–55
	Posttest	132	46‐05 (7.6)	21–61
WAI	Pretest	129	37.12 (7.27)	7–49
	Posttest^a^	63	44.54 (5.61)	17–49

Abbreviations: *M*, mean; *N*, total sample; RDAS, Revised Dyadic Adjustment Scale; *SD*, standard deviation; WAI, working alliance inventory.

^a^ There are less completed WAI questionnaires at posttest since it is scheduled after the patients have had their last couple session, many have therefore assumingly chosen not to fill out the questionnaire.

**Table 2:** Pearson correlations.

	RDAS pretest	WAI pretest	RDAS posttest	WAI posttest
RDAS pretest	–	−0.03	0.43**	0.08
WAI pretest	−0.03	–	0.04	0.36**
RDAS posttest	0.43**	0.04	–	0.16
WAI posttest	0.08	0.36**	0.16	–

Abbreviations: RDAS, Revised Dyadic Adjustment Scale; WAI, Working Alliance Inventory.

** *p* < 0.01.

### Test of nonindependence

The dyadic analyses showed that the error variances for the females were slightly higher than the error variances for males when the WAI was considered as the covariate (*e*_1_ = 67.48, *e*_2_ = 61.6, respectively). According to Cohen (1992) the correlation between these error variances may be assessed as large (*d* = 0.59, *p* < 0.001). When the RDAS was applied as the covariate the results were similar, but the males' error variances were slightly higher than their partners' (*e*_2_ = 30.08, *e*_1_ = 24.08, respectively). The correlation between these scores may be considered medium (*d* = 0.34, *p* < 0.001). These results suggest that these scores between dyad members are nonindependent.

### The RDAS as the outcome variable with the WAI as the process variable

The results of the dyadic analyses show that there was a main effect of gender on the intercept of the RDAS (estimates of 40.28, *p* < 0.001, and 42.73, *p* < 0.001 for females and males, respectively). Our test statements show that the difference between these estimated scores on the intercept was significant (estimate 2.45, *p* < 0.001). There was also a significant and positive main effect of time on the estimated RDAS scores (estimate 0.31, estimate *p* = 0.005).

There was a significant negative actor effect regarding the males' WAI scores' influence on the RDAS scores from one week to the next (estimate −0.31, *p* < 0.001). Further, there was a positive partner effect of the males' WAI scores' influence on the females' RDAS scores from one week to the next (estimate 0.25, *p* = 0.001). These gendered actor and partner effects were further supported by the test statements (estimate −0.34, *p* = 0.002, and estimate 0.22, *p* = 0.042, respectively). The average actor and partner effects according to the test statements were also significantly different between genders (estimate −0.14, *p* = 0.007, estimate 0.14, *p* = 0.007, respectively).

### The WAI as the outcome variable and the RDAS as the process variable

The results of the dyadic analyses show that there was a main effect of gender on the intercept of the WAI (estimate 42.48, <0.001 and estimate 41.97, *p* < .001 for females and males, respectively). Our test statements show that the difference between these scores on the intercept was nonsignificant (*p* = 0.1). There was a significant positive main effect of time on the estimated WAI scores (estimate 0.22, *p* = 0.003).

There was a significant negative actor effect of the females' RDAS scores' influence on the WAI scores from one week to next (estimate −0.18, *p* < 0.001). There was also a significant positive partner effect of the males' RDAS scores on their partners' WAI scores from one week to the next (estimate 0.15, *p* = 0.007). The gender difference for the actor effect was further supported by the test statements (estimate 0.16, *p* = 0.018), while the proposed gender difference of the partner effect was nonsignificant (*p* = 0.204). The average actor and partner effects were significantly different between genders (estimate −0.10, *p* = 0.003, and 0.11, *p* = 0.003, respectively). See Table 3 for an overview of the results of the cross‐lagged dyadic analyses.

**Table 3:** Influence of the covariate on the dependent variable.

Dependent variable	WAI	RDAS
Fixed parameters		
Intercept female	42.48** (0.44) [41.61–43.36]	40.28** (0.69) [38.91–41.64]
Intercept male	41.97** (0.45) [41.09–42.86]	42.73** (0.67) [41.42–44.04]
Time	0.22* (0.07) [0.08–0.37]	0.31* (0.11) [0.10–0.53]
Actor effect, female	−0.18** (0.05) [−0.28 to −0.09]	0.03 (0.07) [−0.11 to 0.16]
Actor effect, male	−0.02 (0.05) [−0.12 to 0.07]	−0.31** (.08) [−0.47 to −0.15]
Partner effect, female	0.06 (0.04) [−0.02 to 0.15]	0.02 (0.07) [−0.12 to 0.17]
Partner effect, male	0.15* (0.06) [0.04–0.26]	0.25** (0.08) [0.1–0.4]
*e*_1_	24.08** (1.65) [21.05–27.54]	67.48** (4.6) [59.04–77.12]
*e*_2_	30.08** (2.08) [2.27–34.44]	61.6** (4.18) [53.93–70.37]
*r*	0.34** (0.05) [0.25–0.43]	0.59** (0.04) [0.52–0.65]

*Note*: The covariate for the RDAS is the WAI and v.v. *e*_1_ = The variance of the female; *e*_2_ = The variance of the male; *r*  = the correlation between *e*_1_ and *e*_2_ and is a measure of nonindependence; 95% confidence interval is given in brackets.

Abbreviations: RDAS, Revised Dyadic Adjustment Scale; WAI, Working Alliance Inventory.

* *p* < 0.01;

** *p* < 0.001.

## DISCUSSION

The purpose of the present study was twofold: Partly to present and exemplify how dyadic analysis and the APIM may be applied to longitudinal data, and to further examine how the working alliances between clients and therapists are associated with couple satisfaction. Our results indicate that the estimated scores of dyad members were nonindependent, ranging from medium (the WAI as the dependent variable) to large (the RDAS as the dependent variable) effect sizes (Cohen, 1992). Given these results we should be able to induce that scores from members of the same dyad were interdependent on one another, indicating that the conditions for interpreting actor and partner effects were met.

As the results of the analysis show dyads improved on both measures of the alliance and couple satisfaction as an effect of time, this finding coincides with the literature on the effectiveness of couple therapy (Barbato & D'Avanzo, 2020; Heatherington et al., 2015; Roddy et al., 2020; Shadish & Baldwin, 2003). Of greater interest are the actor‐partner effects: There was a significant propensity for actor effects to be related to a negative influence on the dependent variable, conversely the partner effects were associated with a positive influence. Specifically, males' estimated alliance scores had a negative influence on their reported couple satisfaction when assessed a week later. For females, the significant actor effect was that their reported couple satisfaction negatively impacted their alliance with their therapist the week after. The test statements supported a gender difference for these actor effects.

Interestingly, both significant partner effects were related to how the males' estimated scores on the WAI and the RDAS positively predicted the females' respective dependent variable (i.e., the RDAS and the WAI) a week later. A stronger alliance between males and their therapist positively predicted the females' reported couple satisfaction, and the more satisfied the males were with their couple relationship yet the stronger their partners' alliances became with their therapist. According to the test statements, support was yielded for gender differences in how the WAI predicted the RDAS, but not conversely.

In general, these results support the body of literature which associates alliance with the outcome (Flückiger et al., 2018; Friedlander et al., 2018), but also bring nuance to how we may understand this interplay from a systemic perspective. More specifically regarding couple therapy, these results indicate that there is not a direct path of positive influence between the individual member of a dyad's alliance (i.e., actor effect) with their therapist, and the couples' perceived relationship satisfaction. On the contrary, actor effects seem to have a negative impact on the outcome. Instead, partner effects are those that most positively influenced the individuals' perceived alliance with the therapist and their couple's satisfaction. As our findings indicate: Strengthening of the male alliance in therapy predicts an increase in his spouse's couple relationship satisfaction—suggesting that establishing a strong alliance with the male member of the dyad may play a crucial role in facilitating a positive outcome of couple therapy. This interpretation finds support from previous studies (Halford et al., 2016; Symonds & Horvath, 2004). Alternatively, these indicated gendered effects may possibly be understood as an expression of interactional patterns (e.g., demand‐withdraw or pursuer‐distancer interactions; Betchen & Ross, 2000; Heavey et al., 1995) and/or attachment styles (Hazan & Shaver, 1987; Simpson, 1990).

Due to western culture's inclination towards linear thinking (Yama & Zakaria, 2019) it may be hard to explain why actor effects are associated with negative influence while partner effects are related to positive influences on the dependent variable. The established literature promoting the working alliance as a predictor for the outcome (Flückiger et al., 2018; Friedlander et al., 2018) makes this explanation especially challenging. Initially one may assume that a strong client‐therapist alliance experienced by either member of the dyad would have the same effect on the outcome, but as the results of this study suggest, this is not the case. A way of making sense of the actor‐partner effects identified in this study may be to consider them as a pattern of contrasts. According to Kenny et al. (2020) contrast effects occur when an individual's responses reverse over repeated interactions. One possible explanation for such a pattern is that female participant in our sample had a higher frequency of psychiatric diagnoses than their spouses and were thereby initially perceived as “the identified patient” by their spouses. As the therapy progressed and the male's alliance with the therapist gained in strength, it is plausible that the presented problem was concurrently reframed from a problem understood as being innate to the individual, to a problem being understood in relational terms (i.e., to a problem occurring between them). One may assume that this unfolding of the therapeutic process thus influenced an increased understanding of their own contribution to their relational problems—a precondition for them to fully engage in therapy. Such an interpretation may also explain why the women's actor effects consisted of gained couple satisfaction exerting a negative influence on their alliance with their therapist: As they witnessed their spouse getting more engaged in therapy (i.e., males partner effect of the alliance positively predicted spouses' couple satisfaction) they had less immediate need of support from their therapist. This was reflected in slight but significant decreases in women's alliance scores across time as they became more satisfied with their partners, that is, experiencing greater support from them. In summary, as the therapy progressed the female members' assessment of the alliance with the therapist reversed as the dyads' couple satisfaction concurrently increased (i.e., signifying less dependence upon the therapist and greater trust in their partners). Although this emergent understanding of change constitutes a hypothesis that needs to be tested, we believe it convenes with the systemic assumptions of circularity that underpin couple therapy (Pinsof et al., 2018; Sprenkle et al., 2009). Further, indirect support for this interpretation may be found when assessing the intercepts of dyad members—they indicate that women are more dissatisfied at the start of therapy (i.e., the complainant) while males have a lower initial alliance with their therapist (i.e., the withdrawer). Similar findings have also been documented elsewhere in the literature on couple therapy (Friedlander et al., 2018; Jackson et al., 2014). In elaboration, women are more dissatisfied with their relationships at the start of therapy and are also disproportionally the ones that initiate couple therapy compared to their male counterparts (Boisvert et al., 2011; Doss et al., 2003). Further, evidence also suggests that men often are the ones that experience pressure to attend therapy, which initially may challenge alliance formation with their therapist (Halford et al., 2016). However, recent research has also implicated previous trauma as a variable that negatively influences men's alliance at the beginning of therapy (Anderson et al., 2020).

Overall, the literature seems to align with our interpretation that males' alliance with the therapist in couple therapy seems central to the outcome. Our emergent model of change does not predict males couple satisfaction beyond time in therapy. Most likely there are other factors that contribute such as trauma, family functioning, a decrease of symptoms of mental distress, or increase in wellness not taken into account of in the current research design. Even if future research does not support our interpretation, but rather reveals other variables as mechanisms of change, the actor‐partner effects as presented in this study still indicate that the change individuals and couples undergo in conjoint therapy is highly associated with how their partner responds to therapy.

### Clinical implications

The results of this study suggest that it is especially important for any couple therapist to assure that the male client is engaging with the therapy as this may facilitate the relationship satisfaction of his female spouse. As therapy progresses the therapist should continue to monitor alliances and outcomes and adjust to accommodate the client's needs to the extent that it is in service of the treatment. The application of routine outcome monitoring (ROM) in its various guises has proven to be an effective tool to identify therapies that are off‐track (Lambert & Shimokawa, 2011) and may therefore be applied to monitor the establishment and maintenance of the alliance. Even though client self‐report questionnaires are preferable to direct questioning by the therapist (Tilden & Wampold, 2017), we believe that any effort to handle and maintain the alliance is better than negligence. There exists examples in the literature on how such feedback may be collected verbally in‐session with clients (e.g., “was there a certain event in this session that was more important for you than any other?”; McLeod., 2017), and we encourage adjusting these so they suit couple therapy (e.g., circular questioning; “could you tell your partner what you thought was the most important event in this session for them?”).

### Limitations

Even though this study successfully shows how cutting‐edge statistical methods may be applied to study reciprocal effects, the current research design could have been improved. Since the study aimed to be practice‐oriented, the measurements used were already implemented at the clinic. Thus, the assessment of the alliance is done with the WAI (Horvath & Greenberg, 1989) only. The WAI is not designed to be used in couple therapy and thus lacks questions related to different subsystems of the clients‐therapist system (Pinsof et al., 2008). Implementing systemic measures of alliances and analyzing such data with dyadic analyses within the APIM would be a great advantage over the measurements used in the current study.

There are also several factors related to psychiatric disorders and past histories of trauma that may potentially have impacted the results of this study. We mention this since most of the sample do fulfill the criteria for a psychiatric diagnosis according to the ICD‐10 (World Health Organization, 1992) and at least half of the sample has been exposed to a traumatic experience. From the literature, we know that there is a negative association between mental distress and couple satisfaction (Whisman & Baucom, 2012; Whisman et al., 2000), as well as a suggested link between trauma and alliance formation in couple therapy (Anderson et al., 2020). We are thus cautious in generalizing our findings to other populations. We only included heterosexual couples because gender was the obvious distinguishing variable, thus our findings may not be generalizable to couples with a different sexual orientation. However, since there is evidence for the association between the occurrence of psychiatric symptoms and relationship discord across cultures and ethnicities (McShall & Johnson, 2015), one would anticipate finding variations of the identified pattern of reciprocal effects (i.e., the proposed importance of the partners' responsiveness to therapy) that share commonalities in other comparable populations.

Even though we acknowledge that the uniqueness of the treatment program that is on offer at the FU might limit the generalizability of the results, we propose that the patterns of associations of alliances and couple relationship satisfaction identified would likely be similar in other contexts of treatment (e.g., outpatient). Studies from other contexts on the alliance in couple therapy show similar results as our study, hence supporting such an interpretation (e.g., Anderson et al., 2020; Halford et al., 2016). Lastly, the naturalistic design of this study may also limit generalizability because several indirect effects are not accounted for (e.g., what interventions were implemented by the couple therapist?). Although this is a valid argument, we believe that naturalistic studies using quantitative methods as presented in this study are of value since they do with accuracy reflect how dyads change across time (Kenny et al., 2020).

## CONCLUSION AND FUTURE RESEARCH

The results of this study show how the method of dyadic analysis is applicable to examine phenomena such as alliances and their interplay with couple satisfaction. Our findings indicate that the alliance and couple satisfaction scores of dyad members are highly interdependent. Furthermore, our analysis suggests that engaging the male member of a dyad in the therapeutic process, as measured by the working alliance, seems to be essential for a successful therapeutic outcome. To our knowledge this detailed finding, although familiar to the experienced clinician, is novel within the field of couple therapy research. Our nuanced results are achieved by using dyadic analysis—a statistical approach that is applicable to any researcher who is interested in reciprocal effects when for instance studying couple relationships, co‐parenting, parent‐child transactions, or the therapist‐client working alliance. Future research applying dyadic analysis to already well‐researched phenomena (e.g., therapeutic alliances) and underresearched populations (e.g., ethnic minorities, same‐sex couples, and survivors of trauma) will likely amount to new knowledge of importance and contribute to moving the field of couple and family therapy forward.
